# Depth of Stromal Invasion as the Most Prognostically Relevant Regression System in Locally Advanced Cervical Cancer after Neoadjuvant Treatment: A Systematic Review and Meta-Analysis Grading

**DOI:** 10.3390/diagnostics11101772

**Published:** 2021-09-26

**Authors:** Gian Franco Zannoni, Antonio Travaglino, Antonio Raffone, Damiano Arciuolo, Nicoletta D’Alessandris, Giulia Scaglione, Pietro Tralongo, Frediano Inzani, Giuseppe Angelico, Angela Santoro

**Affiliations:** 1 Unità di Ginecopatologia e Patologia Mammaria, Dipartimento Scienze della Salute della Donna, del Bambino e di Sanità Pubblica, Fondazione Policlinico Universitario A. Gemelli, IRCCS, Largo A. Gemelli 8, 00168 Roma, Italy; damiano.arciuolo@policlinicogemelli.it (D.A.); ndalessandris@gmail.com (N.D.); scaglione.giulia90@gmail.com (G.S.); pietrotralongo@gmail.com (P.T.); frediano.inzani@policlinicogemelli.it (F.I.); giuangel86@hotmail.it (G.A.); angela.santoro@policlinicogemelli.it (A.S.); 2 Istituto di Anatomia Patologica, Università Cattolica del Sacro Cuore, Largo A. Gemelli 8, 00168 Roma, Italy; 3 Department of Advanced Biomedical Sciences, Pathology Section, School of Medicine, University of Naples “Federico II”, Via Sergio Pansini 5, 80131 Naples, Italy; antonio.travaglino.ap@gmail.com; 4 Gynecology and Obstetrics Unit, Department of Neuroscience, Reproductive Sciences and Dentistry, School of Medicine, University of Naples Federico II, 80131 Naples, Italy; anton.raffone@gmail.com

**Keywords:** pathological response, neoadjuvant setting, cervical cancer, prognosis, meta-analysis

## Abstract

**Background:** several different criteria have been proposed to categorize the pathological response in cervical cancer after neoadjuvant therapy; although it is unclear what the most prognostically valuable one is. **Objective:** to assess the prognostic value of pathological criteria for categorizing the response in cervical cancer after neoadjuvant therapy, through a systematic review and meta-analysis. **Methods:** four electronic databases were searched from January to December 2020 for all studies, assessing the prognostic value of pathological response in cervical cancer after neoadjuvant therapy. Hazard ratio (HR) for overall survival (OS) was calculated with a significant *p*-value < 0.05. A meta-analysis was performed for each criteria assessed in at least three studies. **Results:** sixteen studies were included. Criteria for pathological response included (i) residual stromal invasion < vs. >3 mm; (ii) complete response vs. any residual; (iii) proportion of viable cells; (iv) residual tumor diameter; and (v) intracervical vs. extracervical residual. Criteria (i) and (ii) were suitable for meta-analysis. The presence of a residual tumor with stromal invasion > 3 mm showed a HR of 4.604 (95% CI; 3.229–6.565; *p* < 0.001), while the presence of any residual showed a HR of 1.610 (95% CI; 1.245–2.081; *p* < 0.001); statistical heterogeneity was absent in both analyses. **Conclusions:** dichotomizing the pathological response in cervical cancer after neoadjuvant therapy as < vs. >3 mm stromal invasion is more prognostically valuable than dichotomizing as complete response vs. any residual. Further studies are necessary to evaluate other systems.

## 1. Introduction

Globally, cervical cancer continues to be one of the most common malignancies among females worldwide. In 2018, there were an estimated 569,847 new cases and 311,365 deaths worldwide [1]. Among the numerous clinical and pathological prognostic factors for this neoplasm, the International Federation of Gynecology and Obstetrics (FIGO) stage remains the most important to guide therapeutic strategies, with early cervical cancers treated by surgery alone and more advanced forms dealt with combined modality therapies [2,3]. In particular, locally advanced cervical cancer (LACC) is defined as a great size tumoral mass, including International Federation of Obstetrics and Gynecology (FIGO) stages IB2-IVA. Concurrent neoadjuvant unimodal or multimodal therapies (NACT) followed by surgery have been proposed as possible therapeutical approaches for LACC, with the goals of: (1) down-staging the tumor mass to improve operability; and (2) inhibiting metastasis [4]. The effects of preoperative treatment can be histologically evaluated, and the assessment of neoplastic response to neoadjuvant treatments should be integrated in the pathology reports of resection specimens [5]. In cervical cancers, several tumor regression scoring systems were applied; however, no consensus was observed concerning morphological data, and there was no agreement among the different grading systems. Generally, therapy inducted regressive changes may result in different amounts of residual tumors, up to the complete disappearance of malignant cells, with replacement by fibrous or fibro-inflammatory granulation tissue [6]. Moreover, histopathological determination of tumor regression provides important prognostic information and harbors the potential to guide the clinician to proceed with possible additional therapeutic regimens in the postoperative setting [5]. The aim of the present study was to establish a scoring system, to assess cervical cancer pathological response to neoadjuvant treatment, with the best impact on overall survival (OS) and progression-free survival (PFS).

## 2. Materials and Methods

A systematic review and a meta-analysis were carried out following previous studies [7,8,9]. Each step of the review process was performed by two independent authors, who sought consultation in the case of a disagreement. This study was reported according to the PRISMA statement [10].

### 2.1. Search Strategy and Study Selection

Four electronic databases (PubMed, Scopus, Wed of Science, and Google Scholar) were searched from January to December 2020. The following word combination was used: cervical AND (cancer OR carcinoma) AND neoadjuvant AND pathological response. All studies assessing pathological response in cervical cancer after neoadjuvant treatment were included. Inclusion criteria were: extractable hazard ratio (HR) with 95% confidence interval (CI) for the impact of pathological response on overall survival (OS) and progression-free survival (PFS), or available individual data to calculate HR with 95% CI. Exclusion criteria were: <10 patients, studies including less than 10 patients; studies with less than 2 deaths at follow-up; patient cohort already included in previous studies (unless the overlapping studies adopted different pathological criteria to evaluate the response).

### 2.2. Data Extraction

PICO of our study were: P (population) = women with locally advanced cervical carcinoma; I (intervention, risk factor) = optimal pathological response to neoadjuvant treatment; C (comparator) = suboptimal or no pathological response to neoadjuvant treatment; O (outcome) = OS and PFS [10]. The main data extracted were HR with 95% for the impact of pathological response on OS and PFS when available, the results of the multivariate analysis were used. If HR with 95% was not reported, but individual data were available, pathological response, follow-up time, and status (alive vs. dead or recurred vs. disease-free) were extracted for each patient.

### 2.3. Risk of Bias Assessment

The risk of bias within studies was assessed by using the QUADAS-2 as a basis, as previously described [11,12]. For the “patient selection” domain, we assessed whether patient selection criteria and period of enrollment were clearly reported; for the “index test” domain, we assessed whether criteria to evaluate pathological response were clearly reported; for the “reference standard” domain, we assessed whether oncologic outcomes (i.e., OS, PFS) were clearly reported; for the “flow and timing” domain, we assessed whether patients were followed for at least 2 years to evaluate oncologic outcomes. The risk of bias was categorized as “low”, “high”, or “unclear” for each domain, as previously described [13].

### 2.4. Data Analysis

Each criterion to evaluate pathological response was considered suitable for meta-analysis if it was assessed in at least 3 studies. HRs with 95% CI for each study were pooled by using a random effect model; results were reported on forest plots. A *p*-value < 0.05 was considered significant. Statistical heterogeneity among studies was assessed as previously described [14]. For the studies that reported individual data, HR with 95% CI was calculated by performing a Cox regression survival analysis. Data analysis was performed by using Statistical Package for Social Science (SPSS) 18.0 package (SPSS Inc., Chicago, IL, USA) for analyzing data of individual studies and comprehensive meta-analysis (Biostat, 14 North Dean Street, Englewood, NJ 07631, USA) for pooling results.

## 3. Results

### 3.1. Study Selection

**Figure 1:**
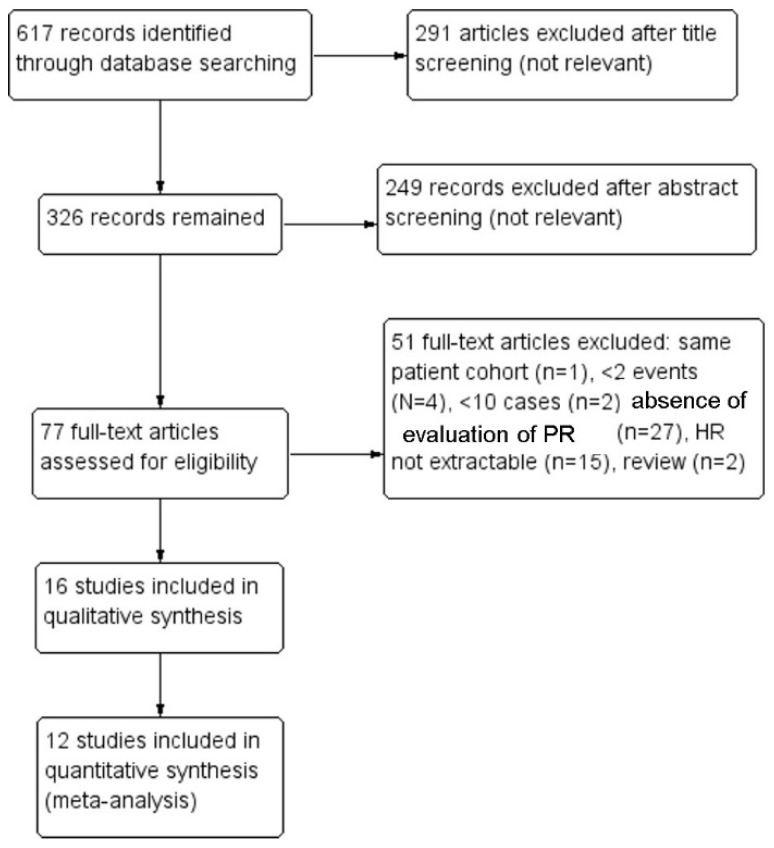
Flow diagram of studies identified in the systematic review (Preferred Reporting Item for Systematic Reviews and Meta-analyses (PRISMA) template). PR—pathological response.

Sixteen studies were included in the systematic review [15,16,17,18,19,20,21,22,23,24,25,26,27,28,29,30] and 12 studies in the meta-analysis [15,16,17,18,19,20,23,24,25,27,28,29,30]. Fifty-one studies assessed for eligibility were excluded after applying our exclusion criteria, i.e., the same cohort as a study already included across multiple papers (n = 1), <2 events (*N* = 4), <10 cases (n = 2), evaluation of clinical rather than pathological response (n = 27), HR with 95% CI not extractable (n = 15), review (n = 2). The process of study selection is summarized in Figure 1.

### 3.2. Study Characteristics

Eight studies (50%) dichotomized pathological response as “optimal” (complete response or residual with <3 mm stromal invasion) vs. “suboptimal” (residual with >3 mm stromal invasion) [17,19,21,23,24,25,26,29]; two studies (12.50%) assessed the proportion of viable cells (<1/3, 1/3-to-2/3 or >2/3) [15,20]; two studies (12.50%) assessed the diameter of the residual tumor (cut-offs at 1 mm or 5 mm) [16,18]; two studies (12.50%) dichotomized pathological response as complete disappearance of the tumor vs. any residual [23,30]; one study (6.25%) dichotomized the residual tumor as intracervical vs. extracervical [27]; the remaining study (6.25%) adopted several systems simultaneously, proposing a combined score [22]. Seven studies (43.75%) assessed OS [15,16,17,18,19,20,30], one (6.25%) assessed PFS [24], and eight (50%) assessed both OS and PFS [21,22,23,24,25,26,27,28,29]. Characteristics of the included studies are reported in Table 1.

### 3.3. Risk of Bias Assessment

**Figure 2:**
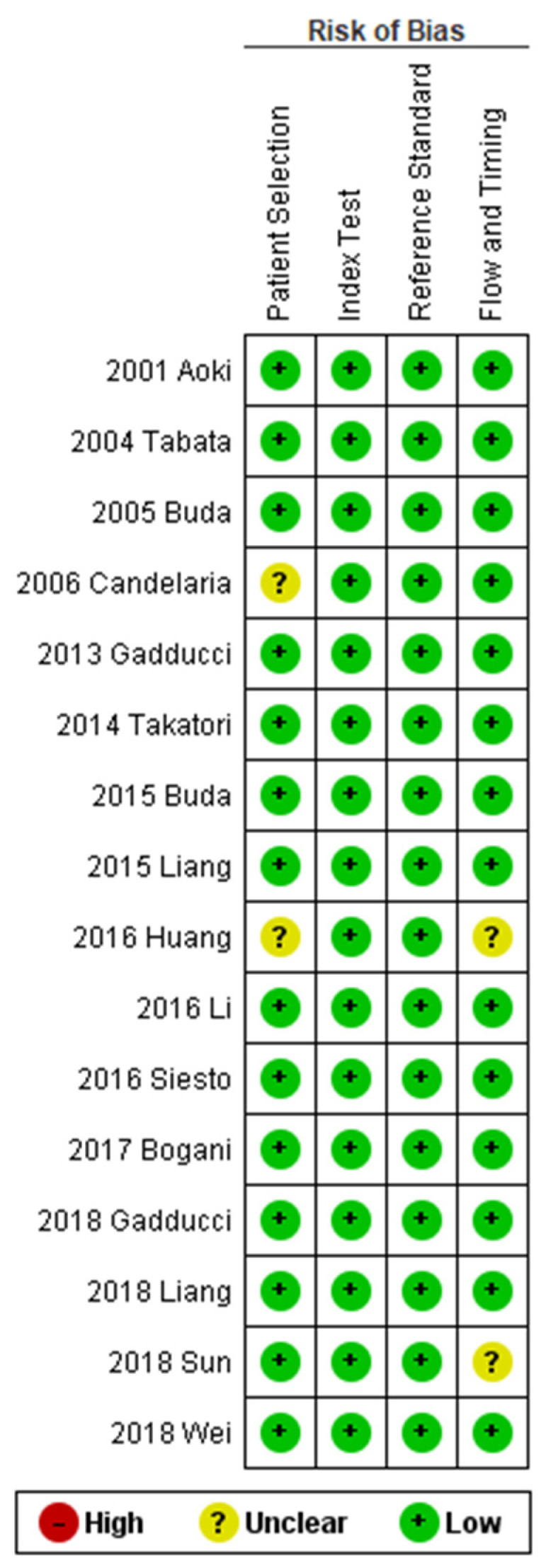
Assessment of risk of bias. Summary of risk of bias for each study; plus sign: low risk of bias; minus sign: high risk of bias; question mark: unclear risk of bias.

The risk of bias was unclear in two studies (12.50%) for the “patient selection” domain (since they did not report the period of enrollment) [18,23] and in two studies for the “flow and timing domain” (since they did not report the follow-up duration) [23,29]; the other studies were considered at low risk of bias for these domains. For the “index test” and “reference standard” domain, all studies were considered at low risk of bias, since they clearly reported the criteria for pathologic response and the oncologic outcomes, respectively (Figure 2).

### 3.4. Meta-Analysis

**Figure 3:**
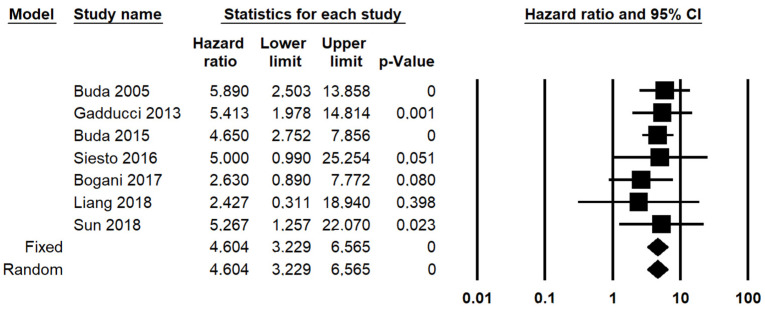
Forest plot reporting hazard ratio (HR) for overall survival in patients with cervical cancer treated, underwent neoadjuvant therapy, obtained by dichotomizing pathological response as “residual tumor with >3 mm stromal invasion” vs. “residual tumor with <3 mm stromal invasion”.

**Figure 4:**
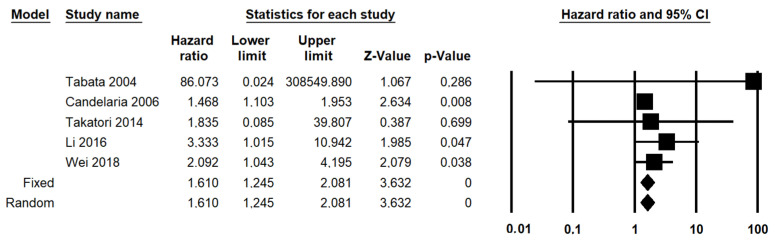
Forest plot reporting hazard ratio (HR) for overall survival in patients with cervical cancer treated, underwent neoadjuvant therapy, obtained by dichotomizing pathological response as “any residual tumor” vs. “complete disappearance of the tumor”.

Two different criteria for categorizing the pathological response were suitable for the meta-analysis of OS: the depth of residual stromal invasion (<3 mm vs. >3 mm) and the presence or absence of the residual tumor (complete disappearance vs. any residual). In fact, data regarding a complete disappearance of the tumor were also extractable from three studies (18.75%) that used different criteria (two that assessed the diameter of the residual tumor [16,17,18] and one that assessed the proportion of viable cells [13]. Only the depth of residual stromal invasion was suitable for meta-analysis of PFS, precluding the possibility of comparisons among different systems. The presence of a residual tumor with stromal invasion > 3 mm was significantly associated with decreased OS, with a HR for OS of 4.604 (95% CI, 3.229–6.565; *p* < 0.001) (Figure 3); statistical heterogeneity among studies was null in both analyses (I^2^ = 0%). The presence of any residual was significantly associated with decreased OS, with a HR of 1.610 (95% CI, 1.245–2.081; *p* < 0.001) (Figure 4), with no statistical heterogeneity among studies (I^2^ = 0%).

## 4. Discussion

Despite the adopted wide-scale cytological screening programs for early diagnosis based on the Pap and/or HPV test, the introduction of the vaccines against HPV infection, and the increased use of condoms during sexual intercourse—cervix cancer is still a public health problem worldwide [4,31]. For locally advanced neoplasms, concomitant definitive chemoradiotherapy (CCRT) is the gold standard therapy, especially in high-risk patients [32,33]. However, different therapeutical choices in the management of LACC are considered worldwide, with the aim of avoiding a significant increase in morbidity and no evident impact on patient survival related to the combination of radical surgery and postoperative external radiotherapy. In fact, in select cases, in absence of negative risk factors, primary radical hysterectomy with intraoperative assessment of lymph node status or paraaortic lymph node dissection for staging purposes is a possible option; neoadjuvant chemotherapy (NACT) followed by radical surgery and/or adjuvant treatments is a controversial alternative to traditional therapies, although it seems comparable to concurrent CCRT [32,33]. Therefore, the assessment of residual disease is required in the pathological report. Notably, regression grading systems mostly refer to the amount of residual neoplastic cells, the induced fibrosis in relation to the residual tumor, or the estimated percentage of the residual tumor compared to the former tumor site. Moreover, a correct pathological restaging has a huge impact on prognosis, follow-up, and/or additional tailored therapies.

A variety of different histological tumor regression scoring systems has been proposed in literature for cervical cancer, but currently, there is no common standard for processing resection specimens after neoadjuvant treatment and for reporting tumor regression. A four-tiered tumor regression score was proposed (levels 0–3) according to the entity of the cancer cell involvement by treatment-induced tumor degeneration or necrosis [34]. Thus, in this study, a significant association with OS was found in the univariate (but not in the multivariate) analysis [34]. Similarly, Takatori E et al. considered four levels of response (Grade 0–3) according to the entity of the residual viable tumor cells [20]. According to the tumor necrosis rate (TNR), the presence and the extent of microscopic coagulative necrosis should be considered another possible system to categorize the pathological response. However, the TNR failed to be a prognostic indicator for patients affected by cervical carcinoma treated with neoadjuvant therapy [35]. In 2008 a three-tiered tumor pathological regression (pR0-2) system considered three types of pathological response, according to the presence/absence of the residual tumor and its diameter (> or <0.3 cm [6]. This scoring system demonstrated a strong relationship between pathological response and morphological changes. The depth of invasion of the residual seemed to be irrelevant, as a measure of aggressiveness [6]. In fact, in their study, the authors found an isolated group of cells (pR1) deeply infiltrating (>0.5 cm), and pR2 residual tumors infiltrating less than 0.5 cm. On the other hand, parametria infiltration was observed only in pR2 cases [6]. Other authors assessed the pathological response as follows: PCR, pathological complete response, as the complete disappearance of the tumor from the cervix and nodes; PR1, partial response 1, as the residual disease with less than 3 mm stromal invasion, including in situ carcinoma with or without lymphatic metastasis; and PR2, partial response 2, as persistent residual disease with more than 3 mm stromal invasion in the surgical specimen [23]. Studies chose 3 mm as the lowest limit of optimal pathological response (PCR + PR1) because it represents the maximal extension of FIGO stage IA1 cervical cancer [17,19]. Optimal pathological response in the cervix was demonstrated to be related to better survival, so that it may serve as a useful prognostic indicator for cervical cancer patients who received neoadjuvant chemotherapy [23]. Only one study has adopted the Mandard tumor regression grade 5-tiered system for the pathological regression scoring in cervical cancer, referring to the amount of therapy-induced fibrosis in relation to the residual tumor [35]. Grade 1 (complete regression) showed absence of histologically identifiable residual cancer and diffuse fibrosis with or without granuloma. Grade 2 was characterized by the presence of rare residual cancer cells scattered through the fibrosis. Grade 3 induced an increase in the number of residual cancer cells, but fibrosis still predominated. Grade 4 showed residual cancer outgrowing fibrosis. Grade 5 was characterized by the complete absence of regressive changes. However, the authors considered the tumor regression grade as a subjectively poor reproducible criterion not based on an objective measurement, and their results showed no clear prognostic significance. Other studies assessed the diameter of the residual tumor with cut-offs at 1, 3, or 5 mm, without reporting significant prognostic values for these thresholds [16,18,22]. Categorization into intracervical vs. extracervical residual disease was also proposed, showing significant association with both OS and PFS [19]. Finally, many authors simply dichotomized pathological response as a complete disappearance of the tumor vs. any residual. From a clinical perspective, pre-operative clinical FIGO staging as well as the WHO double diameter measurement evaluation and RECIST 1.1 criteria, according to tumor size, assessed by transvaginal ultrasound examination, are considered useful predictive indicators of patient outcomes [36]. In particular, WHO criteria and Response Evaluation Criteria in Solid Tumors (RECIST 1.1) are widely used and based on the presence of clinically measurable lesions. These methods clearly define the terms of complete response (CR), partial remission (PR), progressive disease (PD), and stable disease (SD). Patients with CR or PR were classified as clinical responders, and patients with SD and PD were defined as clinical non responders [34]. However, given the fact that treated cervix cancers did not always have measurable lesions, a pathological evaluation would probably have a better correlation with prognosis than RECIST criteria. Among all of these clinical and pathological tumoral regression scoring methods, it is still a matter of debate which system provides the best interobserver agreement or the most prognostic value. In our study, only two different criteria for categorizing pathological response were suitable for meta-analysis of OS: the depth of residual stromal invasion (<3 mm vs. >3 mm) and the presence or absence of residual tumor (complete disappearance vs. any residual). Only the depth of residual stromal invasion was suitable for meta-analysis of PFS, precluding the possibility of comparisons among different systems. We found that the presence of the residual tumor with stromal invasion >3 mm had a HR for OS of 4.604, while the presence of any residual had an HR of 1.610; the 95% CIs did not overlap, indicating a statistically significant difference between the two. These results support the notion that a criterion of 3 mm of residual stromal invasion might represent an optimal prognostic stratification strategy, better than labeling any residual tumor as a suboptimal response. Our findings show that the pathological evaluation of the residual tumoral invasion depth is highly valuable in stratifying prognosis of LACC after neoadjuvant treatment, suggesting that a residual with <3 mm stromal invasion is prognostically similar to a complete absence of any residual tumor. Such system appears strengthened by the objectivity of the measurement of the depth invasion, which would make it more reproducible than systems based on the proportion of necrotic/degenerative changes. Further studies are necessary to evaluate the prognostic value of other systems to categorize pathological response of LACC to neoadjuvant treatment.

## 5. Conclusions

Implementing a tumor regression scoring method to cervix cancer appears relevant; neoadjuvant therapy has emerged as a promising step forward in the management of LACC. In line with previous studies, this meta-analysis confirms that evaluating pathological response based on the residual depth of invasion has a high prognostic value in terms of OS. Such an approach appears objective, easily applicable, reproducible, and is based on quantitative measurable parameters. We are aware that further studies should investigate and compare the prognostic impacts of the different clinical and pathological tumor regression scoring systems. Whether complete tumor response indicates a peculiar biological characteristic, as an incapability of developing tumor cell clones resistant to therapy, or is an effect of optimal treatment, remains to be determined in future research, likely at a molecular level.

## Figures and Tables

**Table 1:** Characteristics of the included studies. *: Available data for complete disappearance vs. any residual. **: 25th and 75th percentile are reported instead of range.

Study	Country	Institution	Period of Enrollment	Sample Size	Histotype	Stage	Evaluation of Pathological Response	Oncologic Outcome Assessed	Follow-up Duration, Mean/Median (Range)
Aoki 2001 [14]	Japan	Niigata University Hospital	1993–1998	11	AC	IB-III	proportion of viable cells (no vs. <1/3 vs. 1–2/3 vs. >2/3)	OS	30 (1–65) m
Tabata 2004 [15]	Japan	Mie University	1997–2002	14	AC	IB-IIB	residual lesion size (no vs. <5 mm vs. >5 mm) *	OS	47 (14–83) m
Buda 2005 [16]	Italy	21 Italian centers	1997–2000	219	SCC	IB2-IVA	residual stromal invasion depth (<3 mm vs. >3 mm)	OS	43 (31–56) m **
Candelaria 2006 [17]	Mexico	Instituto Nacional de Cancerología	unclear	178	AC, SCC	IB2-IIIB	residual lesion size (no vs. <1 mm vs. >1 mm) *	OS	30 (3–66) m
Gadducci 2013 [18]	Italy	− University of Turin − University of Pisa − University of Brescia − European Institute of Milan	2002–2011 2009–2011	333	AC, SCC	IB2-IIB	residual stromal invasion depth (<3 mm vs. >3 mm)	OS, PFS	66 (8–212) m
Takatori 2015 [19]	Japan	− Iwate Medical University − National Hospital Organization Kokura Medical Center − Miyama Hospital, Oshu − Medical Coat Hachinohe West Hospital	2002–2012	33	SCC	IB2-IIB	proportion of viable cells (no vs. <1/3 vs. 1–2/3 vs. >2/3) *	OS	34 (6–112) m
Buda 2015 [20]	Italy	San Gerardo Hospital of Monza	1992–2011	446	AC, SCC	IIB-IVA	residual stromal invasion depth (<3 mm vs. >3 mm)	OS	152 (98–193)**
Liang 2015 [21]	China	Women’s Hospital, Zhejiang University	2003–2012	204	SCC	IB2-IIA	-proportion of viable cells -residual stromal invasion depth -residual lesion size	OS, PFS	64 (26–128) m
Huang 2016 [22]	China	8 Chinese centers	unclear	853 (retrospective) 603 (prospective)	Any	IB2-IIB	residual stromal invasion depth (<3 mm vs. >3 mm)	PFS	unclear
Li 2016 [23]	China	Sun Yat-sen Memorial Hospital	2005–2010	347	AC, SCC	IB2-IIA	any residual lesion (no vs. yes)	OS, PFS	37 (4–65) m
Siesto 2016 [24]	Italy	Humanitas Clinical and Research Center, Milan	2009–2015	32	AC, SCC	IB2-IIB	residual stromal invasion depth (<3 mm vs. >3 mm)	OS, PFS	36 (5–71) m
Bogani 2017 [25]	Italy	National Cancer Institute, Milan	1990–2011	275	Any	IB2-IIB	residual stromal invasion depth (<3 mm vs. >3 mm)	OS, PFS	48 (not reported) m
Gadducci 2018 [26]	Italy	− University of Turin − University of Pisa − European Institute of Milan	1992–2014	82	AC	IB2-IIB	residual disease extent (intracervical vs. extracervical)	OS, PFS	89 (5–208) m
Liang 2018 [27]	China	Women’s Hospital, Zhejiang University	2007–2014	137	SCC	IB2-IIA	residual stromal invasion depth (<3 mm vs. >3 mm)	OS, PFS	51 (22–117) m
Sun 2018 [28]	China	Huazhong University of Science and Technology, Wuhan	1999–2008	393	SCC	IB2-IIB	residual stromal invasion depth (<3 mm vs. >3 mm)	OS, PFS	unclear
Wei 2018 [29]	China	The Fourth Military Medical University	2009–2014	410	AC, SCC	IB2-III	any residual lesion (no vs. yes)	OS	51 (4–97) m

## Data Availability

All data are available from the corresponding author upon reasonable request.
